# Genomic surveillance of post-immunization pneumococcal carriage in Ghana

**DOI:** 10.1099/mgen.0.001734

**Published:** 2026-06-17

**Authors:** Samuel A. Akwetey, Standford Kwenda, Humphrey P. K. Addy, Ebenezer Foster Nyarko, Elvis Fiam, Elvis Quansah, Irene E. Donkor, Desmond O. Acheampong, Godwin Kwakye-Nuako, Mohammed R. Abdullah, Sven Hammerschmidt, Arshad Ismail, Richael O. Mills

**Affiliations:** 1 Department of Clinical Microbiology, School of Medicine, University for Development Studies, Tamale, Ghana; 2 Centre for Healthcare-Associated Infections, Antimicrobial Resistance and Mycoses, National Institute for Communicable Disease, Johannesburg, South Africa; 3 Department of Biomedical Science, School of Allied Health Sciences, University of Cape Coast, Cape Coast, Ghana; 4 Department of Infection Biology, London School of Hygiene & Tropical Medicine, Keppel Street, London, UK; 5 Department of Biological Sciences Education, Akenten Appiah-Menka University of Skills Training and Entrepreneurial Development, Asante Mampong, Ghana; 6 Department of Medical Laboratory Technology, School of Allied Health Sciences, University of Cape Coast, Cape Coast, Ghana; 7 Institute of Clinical Chemistry and Laboratory Medicine, University Medicine Greifswald, 17475 Greifswald, Germany; 8 Department of Molecular Genetics and Infection Biology, Interfaculty Institute for Genetics and Functional Genomics, Center for Functional Genomics of Microbes, University of Greifswald, 17487 Greifswald, Germany; 9 Sequencing Core Facility, National Institute for Communicable Disease a Division of the National Health Laboratory Service, Johannesburg 2131, South Africa; 10 Department of Biochemistry and Microbiology, Faculty of Science, Engineering and Agriculture, University of Venda, Thohoyandou 0950, South Africa; 11 Institute for Water and Wastewater Technology, Durban University of Technology, Durban 4000, South Africa

**Keywords:** antimicrobial resistance, carriage, Ghana, Global Pneumococcal Sequence Clusters, *Streptococcus pneumoniae*

## Abstract

*Streptococcus pneumoniae* remains a major cause of morbidity and mortality worldwide, especially among children under 5 years of age. The introduction of pneumococcal conjugate vaccines (PCVs) has significantly reduced invasive pneumococcal disease globally. Ghana introduced routine immunization with PCV13 in 2012; however, its impact on pneumococcal carriage at the genomic level remains largely unexplored. This is the first genomic characterization of pneumococcal carriage in Ghana post-PCV13 introduction; in the absence of a matched pre-vaccination genomic dataset, findings are interpreted in the context of the post-vaccination era rather than as a direct pre–post-comparison. We identified substantial genomic diversity among carriage isolates, including 49 Global Pneumococcal Sequence Clusters (GPSCs) and 67 sequence types (STs), of which 21 STs were novel, and 4 were international Pneumococcal Molecular Epidemiology Network clones (Spain9V-3, Greece6B-22, NorwayNT-42 and Sweden15A-25). GPSC 5 (14%) was the most prevalent cluster, followed by GPSC 20 (6%). GPSCs associated with antibiotic resistance (GPSCs 1, 6 and 10) represented 2%, 3% and 2% of isolates, respectively. Importantly, we observed a significant decline in vaccine serotypes (VTs) and a corresponding rise in non-vaccine serotypes (NVTs), notably serotypes 23B and 15BC. Additionally, VTs demonstrated higher levels of antibiotic resistance compared to NVTs (*P*<0.05). This study provides the first genomic epidemiological insights into pneumococcal populations in Ghana post-PCV13 introduction and underscores the importance of continuous genomic surveillance to monitor serotype replacement and antibiotic resistance trends.

**Impact Statement**

The introduction of pneumococcal conjugate vaccines (PCVs) has significantly reduced the burden of invasive pneumococcal disease worldwide. This study investigates the genomic impact of PCV13 on *Streptococcus pneumoniae* carriage in Ghanaian children under 5 years of age. The first genomic surveillance of post-immunization pneumococcal carriage in Ghana provides critical insights into the evolving landscape of *S. pneumoniae* following the introduction of PCV13. Whole-genome sequencing revealed substantial genetic diversity among isolates, identifying 49 Global Pneumococcal Sequence Clusters and 67 multilocus sequence types, including 4 Pneumococcal Molecular Epidemiology Network clones (antibiotic-resistant lineages) and novel sequence types. The findings highlight the continued decline of vaccine serotypes and the concerning rise of non-vaccine serotypes, emphasizing the necessity for ongoing genomic monitoring to track serotype replacement and antimicrobial resistance trends. The data presented offer a valuable framework for informing public health strategies, guiding vaccine policy adaptations and reinforcing antimicrobial stewardship efforts in Ghana and beyond.

## Data Summary

Novel whole-genome sequences of *Streptococcus pneumoniae* were deposited at National Center for Biotechnology Information and accessed under BioProject PRJNA1100994 . The accession numbers are from SRR29842283 to SRR29842319 and from SRR34351849 to SRR34351981. The phylogenetic snapshot of carriage isolates from Ghana is available at https://microreact.org/project/f6bsmGrF9UEM4Y1Ve1D3Z3-pneumophylogh. The authors confirm that all supporting data, code and protocols have been provided within the article or through supplementary data files.

## Introduction

*Streptococcus pneumoniae* is a Gram-positive, lancet-shaped bacterium that colonizes the nasopharynx of ~50% healthy children and 10% healthy adults [1]. Nonetheless, among immunocompromised children, *S. pneumoniae* disproportionately causes mild to life-threatening infections such as otitis media, sinusitis, pneumonia, septicaemia and meningitis [2]. In 2021, pneumococcal disease caused an estimated 179,354 deaths (95% UI: 142,347–217,280) among children and adolescents aged under 20 years globally, with children under five bearing the highest burden (age-standardized mortality rate 23.52 per 100,000) [2]. Additionally, pneumococcal disease is estimated to cause ~49,000 child deaths annually [3].

The main virulence factor of *S. pneumoniae* is its polysaccharide capsule, encoded by the extensively variable *cps* genes [4]. The virulence of this pathogen is exacerbated by its acquired ability to exchange genetic material with other members of the genus *Streptococcus* via horizontal gene transfer, enabling continuous acquisition of novel capsule types that drive capsular diversification and immune evasion. This is exemplified by the recent evolution of serotype 20C, which represents, possibly, the 104th identified pneumococcal serotype [5]. In addition to the capsule, virulence factors such as pneumolysin (Ply), pili and zmpC play a role in the colonization of the bacterium [6,7]. Ply, a cytolytic toxin, initiates colonization by promoting inflammatory mucus secretions that propel transmission to new hosts. Pili enhance the initial adherence of the bacterium to host nasopharyngeal epithelial cells by binding to platelet endothelial cell adhesion molecule 1. ZmpC cleaves the chemokine CXCL8/IL-8, thereby impairing neutrophil recruitment and facilitating mucosal colonization and the progression of infection. Ply, pili and ZmpC are of particular interest beyond their roles in colonization and infection: as highly conserved protein antigens present across serotypes, they are under active investigation as candidates for next-generation, serotype-independent pneumococcal vaccines, which may offer broader protection than current polysaccharide conjugate formulations, including in low- and middle-income country settings where serotype diversity is high.

Despite the considerable diversity of pneumococcal serotypes, only a subset is responsible for invasive pneumococcal disease [8]. To mitigate the burden of pneumococcal disease, various pneumococcal conjugate vaccines (PCV) have been implemented in many countries [3]. In 2000, the 7-valent PCV (PCV7), targeting serotypes 4, 6B, 9V, 14, 18C, 19F and 23F, was approved for use in young children in the USA [9]. The introduction of PCV7 in numerous countries has significantly reduced the incidence of invasive pneumococcal disease caused by vaccine-targeted serotypes [10].

A decade after the approval of PCV7, two second-generation, higher-valent PCVs were licensed, replacing PCV7 and broadening serotype coverage. The 10-valent PCV10 (Synflorix^™^, GlaxoSmithKline) added serotypes 1, 5 and 7F to the original PCV7 serotypes (4, 6B, 9V, 14, 18C, 19F and 23F). The 13-valent PCV13 (Prevnar^™^13, Wyeth Pharmaceuticals LLC, a subsidiary of Pfizer Inc.) further expanded coverage by including the PCV10 serotypes along with serotypes 3, 6A and 19A [11,12]. South Africa was among the first African countries to include the PCV into their immunization programme, specifically PCV7, in 2009 and later transitioned to the 13-valent PCV in May 2011 [13]. In May 2012, Ghana incorporated PCV13 vaccination into its childhood immunization regimen, utilizing a 3+0 vaccine schedule. By 2020, the PCV13 coverage in Ghana was estimated at 97%, with a dosing schedule of 6, 10 and 14 weeks [14]; however, coverage varies sub-nationally and may be lower in some regions.

The indiscriminate use of antimicrobial agents in community settings exerts selective pressure that promotes the emergence of resistant pneumococcal strains, leading to treatment failures [15]. Conversely, vaccination imposes selective pressure on the pneumococcal population by reducing carriage of vaccine-types (VT), many of which are associated with antibiotic-resistant lineages. As VT strains decline in prevalence following vaccine introduction, the ecological space they occupied in the nasopharynx becomes available for colonization by non-vaccine-types (NVT) – a phenomenon known as serotype replacement [16]. Since the antibiotic resistance profiles of circulating strains are closely linked to their serotype and clonal background, shifts in the dominant serotypes can substantially alter population-level resistance patterns. In settings where resistant clones are predominantly VT, vaccine-driven displacement may reduce overall resistance burden; however, if expanding NVT lineages acquire or carry resistance determinants, resistance may be maintained or increase despite successful vaccine coverage [16]. Vaccine-driven pressure is associated with serotype replacement via capsular switching, which facilitates the expansion of prevalent strains and the emergence of novel non-vaccine serotypes. Nonetheless, vaccination also reduces the transmission of vaccine-targeted strains, conferring indirect herd immunity to unvaccinated populations [17,18].

Genomic approaches have proven instrumental in our understanding of the biology and epidemiology of important bacterial pathogens such as *S. pneumoniae* [19]. Whole-genome sequencing (WGS) studies offer deeper insights into Global Pneumococcal Sequence Clusters (GPSCs), multilocus sequence types (MLST), defined by variations in housekeeping genes, and their associated clonal complexes (CCs) [16].

Before the introduction of the 13-valent PCV in Ghana, the pneumococcal carriage ranged from 31% to 51.4% [20,22]. Six years after the introduction, carriage rates remained notably high, at 54% [23]. Subsequent studies in 2018 reported carriage rates of 29.4 and 32.6% in Cape Coast and Kassena Nankana, respectively [24,25]. Despite these comparatively low carriage rates, an increase in non-vaccine types was observed, likely due to serotype replacement driven by capsular switching. Importantly, no prior studies have characterized the sequence types (STs) associated with these emerging serotypes, nor have genomic analyses been conducted to identify GPSCs circulating in Ghana.

In this study, we examine the distribution and genetic diversity of local lineages and antimicrobial resistance in pneumococcal isolates obtained from healthy Ghanaian children under 5 years of age following the introduction of PCV13. Utilizing WGS data, we aimed to identify circulating serotypes, assess key virulence factors such as pilus islets and zinc metalloproteinase C and investigate genomic events, including capsular switching. This approach provides critical insights into the impact of PCV13 on pneumococcal populations and antimicrobial resistance patterns in our setting.

## Methods

### Study setting and bacterial isolates

The pneumococcal isolates included in this study were collected during cross-sectional studies conducted in Cape Coast, the capital of Ghana’s Central Region, between January and March 2018. Cape Coast is a coastal city located ~144 km west of Accra, with a predominantly urban population distributed across several distinct communities.

**Fig. 1.**
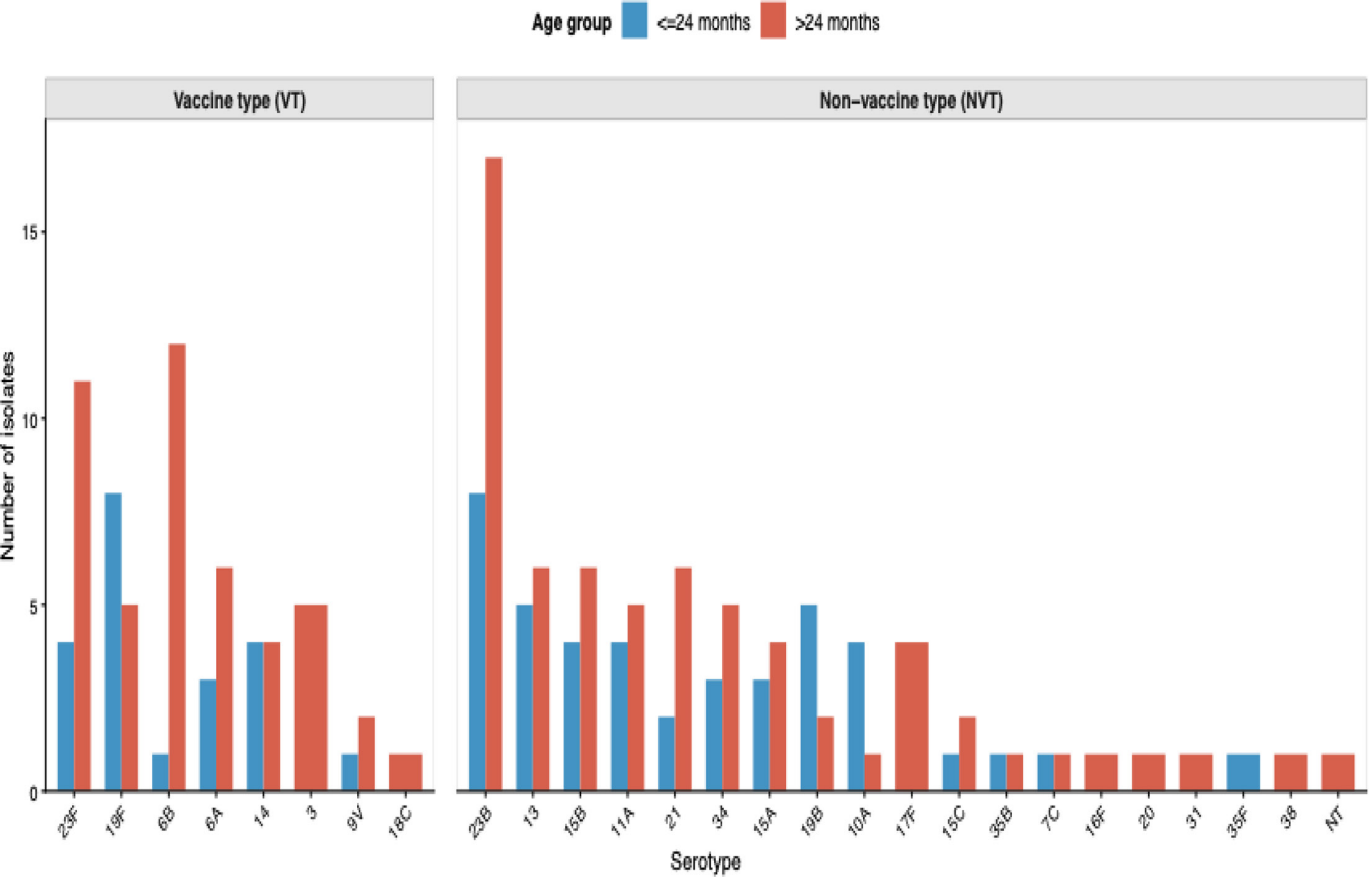
Distribution of pneumococcal serotypes by age group and vaccine type.

Nasopharyngeal swabs were taken from healthy children under 5 years of age with consent from their parents. Participants were enrolled from 18 study sites distributed across the Cape Coast Metropolitan Area, spanning communities, including Abura, Amamoma, Akotokyir, Efutu, Ewim, Kwapro, Pedu and UCC community (Fig. 1): 12 pre-schools (comprising nursery and kindergarten settings, both public and private) and 6 reproductive and child health (RCH) clinics.

Nasopharyngeal swabs were collected using standard flexible nylon-tipped swabs inserted to a depth of ~2–3 cm into the posterior nasopharynx, following WHO-recommended procedures for nasopharyngeal sampling in children. All participating children had completed the full PCV-13 vaccination schedule and exhibited no symptoms of respiratory infection at the time of sampling. Sample collection, storage and pneumococcal identification followed the WHO-recommended guidelines [26].

Briefly, nasopharyngeal swabs were cultured on sheep blood agar supplemented with 5 µg ml^−1^ of gentamicin. Presumptive alpha-haemolytic colonies were confirmed by optochin susceptibility and bile solubility. Confirmed pneumococcal isolates were stored in skim milk-tryptone-glucose-glycerol medium at −80 °C for subsequent analyses.

### Serotyping and antimicrobial susceptibility testing

Antimicrobial susceptibility testing was conducted on all isolates using the disc diffusion method and interpreted according to the Clinical and Laboratory Standards Institute 2020 guidelines. Ten antibiotics were tested: oxacillin (OX), ceftriaxone, cotrimoxazole (COT), levofloxacin (LEV), vancomycin (VA), erythromycin (ERY), tetracycline (TET), linezolid (LZD), clindamycin (DA) and chloramphenicol (CHL). For isolates resistant to OX (>19 mm inhibition zone), indicative of penicillin (PEN) non-susceptible pneumococci, MICs for PEN were determined. PEN resistance was defined as an MIC of ≥2 µg ml^−1^, based on non-meningitis breakpoints. Quality control was performed using *S. pneumoniae* ATCC 49619. Multidrug resistance (MDR) was defined as acquired non-susceptibility to at least one agent in three or more classes of antimicrobial agents [27].

Serotyping was initially performed using multiplex PCR (mPCR) following protocols and primer pairs developed for African samples. Isolates not typable by mPCR underwent further serotyping using the Quellung reaction.

### WGS and analyses

Genomic DNA was extracted using the QIAamp DNA Mini Kit (Qiagen, Hilden, Germany). Multiplexed libraries were prepared using the Illumina DNA Preparation Kit according to the manufacturer’s protocol (Illumina) and sequenced on the Illumina NextSeq platform using 2×150 bp paired-end chemistry at the National Institute for Communicable Diseases, Sequencing Core facility, Johannesburg, South Africa.

Genome assemblies were generated using SKESA (v.2.4.0; https://github.com/ncbi/SKESA) within the JEKESA pipeline (v.1.0.0; https://github.com/stanikae/jekesa), which also performed quality control, read filtering, species identification, contamination detection, genome assembly and sequence typing. Contigs with more than 5% contamination were excluded. STs were assigned via PubMLST (https://pubmlst.org/). All analyses were referenced against the complete *S. pneumoniae* R6 genome sequence (serotype 2; ST 595; GPSC 622; GC content 39.7%; NCBI accession NC003098.1).

Quality control of assembled contigs was evaluated based on coverage depth, GC content and assembled read bases (Fig. S1, available in the online Supplementary Material). The assembled genomes were uploaded to Pathogenwatch (https://pathogen.watch/), where GPSC and serotypes were assigned using PopPUNK’s k-mer clustering method [28] and a reference list of pneumococcal isolates (*n*=42163, version 6) and seroBA (v. 1.0.0) [29], respectively. Genotypic antibiotic resistance profiling was performed using Pathogenwatch and AbritAMR (v1.0.7) [30].

A maximum-likelihood phylogenetic tree was constructed using RaxML (v1.2.2; https://github.com/amkozlov/raxml-ng) based on SNPs extracted from reads aligned to the *S. pneumoniae* R6 reference genome. Recombination regions were identified and masked using Gubbins [31]. The resulting recombination-free phylogeny in Newick format (.nwk) was uploaded to the Microreact web tool (https://microreact.org/project/f6bsmGrF9UEM4Y1Ve1D3Z3-pneumophylogh) for visualization.

Strain-sharing cluster analysis was performed using SNP2Cluster (https://github.com/stanikae/SNP2Cluster/tree/v0.5.3) [32]. Two complementary whole-genome clustering approaches were applied: (i) core-SNP clustering, which groups isolates based solely on pairwise core-genome SNP distances, with a threshold of ≤12 SNPs to define closely related isolates; and (ii) SNP-Epi clustering, which integrates both SNP distances (threshold ≤13 SNPs) and epidemiological metadata (facility, time period) to identify epidemiologically linked groups. Isolates were first assigned to clusters using an improved K-means algorithm with silhouette scoring and 500 bootstraps. Pairwise genetic distances were scaled (normalized to mean 0, sd 1) prior to clustering so that variables with larger raw values did not disproportionately influence cluster assignment. The STs and clusters were visualized per facility, sex and vaccine types in a heatmap and minimum-spanning tree.

### Statistical analyses

Isolates were classified as vaccine serotypes (VT) if they belonged to PCV13-covered serotypes (1, 3, 4, 5, 6A, 6B, 7F, 9V, 14, 18C, 19A, 19F and 23F) or as non-vaccine serotypes (NVT) if they belonged to other serotypes. Lineages were categorized as VT GPSC (containing only PCV13 serotypes), NVT GPSC (containing only non-PCV13 serotypes) or mixed GPSC (containing both VT and non-VT serotypes). Capsular switching was identified when isolates of different serotypes belonged to the same ST. CCs were defined using eBURST, grouping STs sharing at least six of seven identical alleles. Relationships between STs, MDR and serotype switching were visualized using PHYLOViZ 2.0 [33]. Association between variables was assessed using chi-square or Fisher’s exact tests, with *P*-values<0.05 considered significant. Bar charts showing counts of serotypes, STs and GPSCs were generated using GraphPad Prism v. 10.2.0 (https://www.graphpad.com/). R v.4.1.2 (https://www.R-project.org/) using UpSetR and ggplot was used to plot the Upset graph and to visualize the phylogenetic tree. Whole-genome clustering refers to grouping of isolates on the basis of genetic similarity across the entire genome, as implemented here via PopPUNK k-mer clustering to assign GPSCs, providing higher resolution than MLST-based CC assignment. WGS clusters represent groups of closely related bacteria identified by analysing their entire DNA sequence.

## Results

### Demographic characteristics of healthy children under five

A total of 911 nasopharyngeal samples were analysed, collected from children under 5 years old. Among these, 244 (26.7%) samples tested positive for *S. pneumoniae*, and 190 isolates were successfully retrieved from frozen storage. Of these, 174 isolates passed quality control using FASTQC (Fig. S1) following sequencing.

Many isolates were obtained from children above 24 months (*n*=111; 63.8%), pre-school-aged children (*n*=133; 76.5%), males (*n*=90; 51.7%) and children residing in the Abura area (*n*=74; 42.5%) (Table 1).

**Table 1.** Characteristics of the study population

Age group		Frequency	Per cent
	≤24 months	63	36.2
	>24 months	111	63.8
**Type of facility**			
	Pre-school	133	76.5
	RCH	41	23.6
**Sex**			
	Female	84	48.3
	Male	90	51.7
**Location**			
	Aboom	2	1.1
	Abura	74	42.5
	Akotokyir	4	2.3
	Amamoma	25	14.4
	Bakano	1	0.6
	Efutu	24	13.8
	Ewim	7	4.0
	Kotokuraba	1	0.6
	Kwapro	10	5.7
	Pedu	3	1.7
	UCC community	23	13.2
	**Total**	**174**	**100.0**

### Serotype distribution and vaccine coverage

**Fig. 2.**
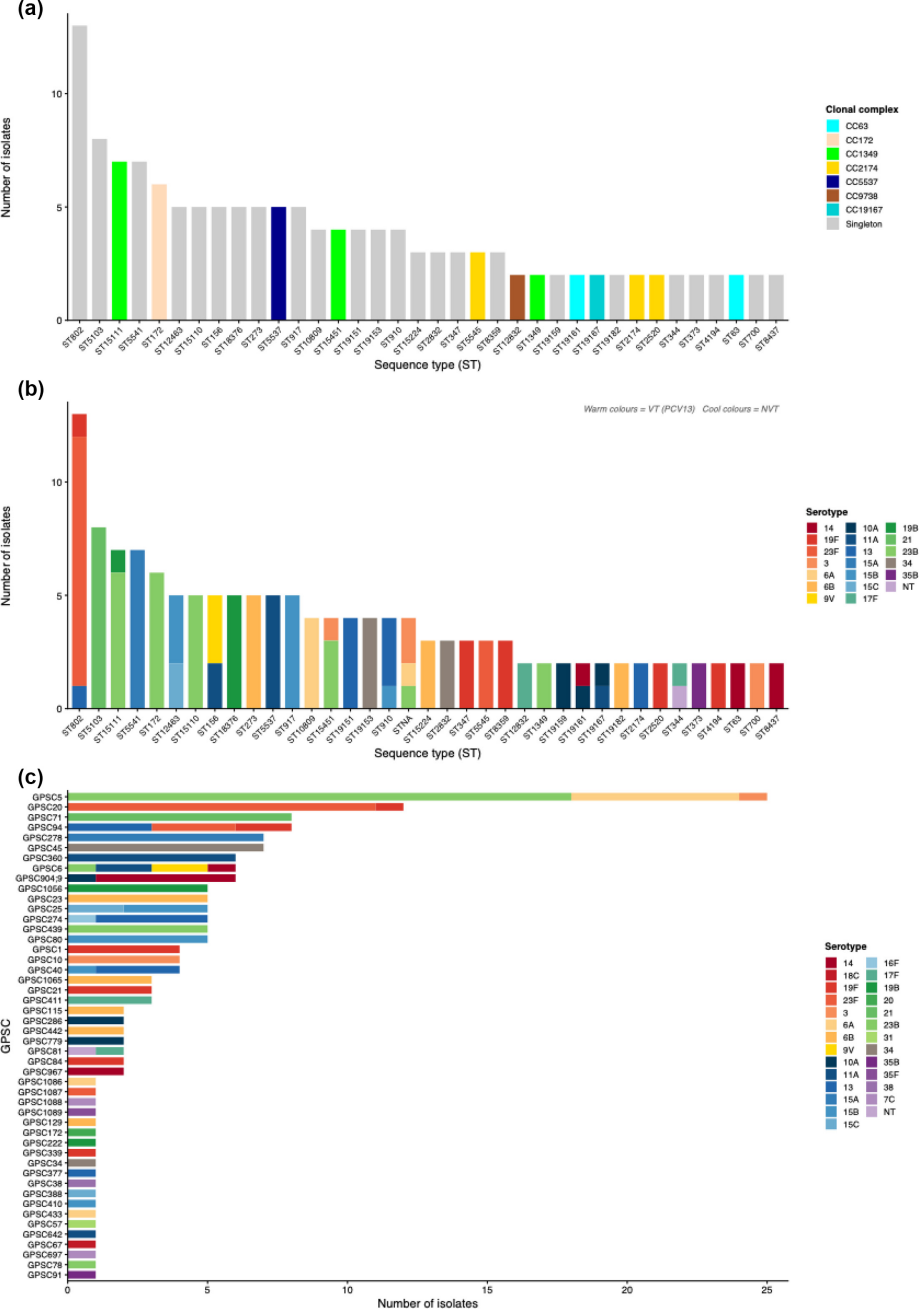
Pneumococcal lineage structure among study isolates (*n*=174).

There was a 100% concordance between phenotypic determination of serotypes using mPCR, the Quellung reaction and genomic serotype predictions. Among the isolates, only 1 was non-typable, while the remaining belonged to 26 distinct serotypes (Fig. 2). Serotypes covered by PCV-13 (vaccine types) accounted for 38.5% of the isolates, whereas non-vaccine types represented 61.5%. PCV-20 expanded coverage to 52.3% of the serotypes, including additional serotypes such as 10A, 11A and 15B.

The most prevalent serotype was 23B (14.4%), followed by 23F (8.6%), 6B (7.5%), 19F (7.5%), 13 (6.3%), 15B (5.7%), 11A (5.2%) and 6A (5.2), indicating the persistence of vaccine types. Most serotypes were detected in both age groups. The least detected serotypes (0.6%) included 35F in children ≤24 months and serotypes 3, 18C, 16F, 20 and 38 in children aged >24 months (Fig. 1).

### Pneumococcal lineages – STs

Sixty-seven (67) distinct STs were identified, including 21 novel STs. These STs were grouped into 8 CCs and 59 singletons at a single locus variant level (Fig. 2a). The CCs showed strong concordance with GPSCs, as detailed in Table S1.

The most prevalent STs were ST802 (*n*=13; 7.5%), ST5103 (*n*=8; 4.6%), ST5541 (*n*=7; 4.0%), ST15111 (*n*=7; 4.0%) and ST172 (*n*=6; 3.4%). A higher proportion of the STs were associated with NVT (55.2%) compared to VT (38.8%) (Fig. 1). Only four STs (0.06%) expressed both serotype groups, suggesting a potential capsular switch between serotype groups. These included the following: ST802, expressing serotypes 23F/19F/13; ST156, expressing serotypes 9V/11A; ST15451, expressing serotypes 23B/3; and ST19161, expressing serotypes 10A/14 (Table S1). Additionally, capsular switching events were observed within vaccine groups, including the following: ST910, expressing serotypes 15B/13; ST12463, expressing 15B/15C; ST15111, expressing serotypes 23B/19B; and ST344, expressing serotypes NT/17F.

### Pneumococcal lineages – GPSCs

The 174 high-quality assemblies were distributed among 49 GPSCs. The most prevalent clusters included GPSC5, comprising CC172, CC1349, ST5549, ST10809 and ST19181 (serotypes 19F/3/23B); GPSC20, associated primarily with ST802 (serotypes 23F/19F); GPSC94, consisting of ST802, and CC2174 (serotype 13); GPSC71, represented by ST5103 (serotype 21); GPSC6, including ST143, ST156 and ST15111 (serotypes 14/11A/9V/23B); GPSC45, comprising ST2825 and ST19153 (serotype 34); and GPSC278, represented by ST5541 (serotype 15A). Collectively, these seven GPSCs accounted for 42.5% of the total isolates analysed (Fig. 2c and Table S1). Notably, 20 of the identified GPSCs were represented by a single isolate each.

Among the identified GPSCs, 17 (35%) consisted exclusively of VT serotypes, 28 (57%) were NVT, and 4 (8%) included both VT and NVT lineages. Mixed lineages included GPSC5, GPSC94, GPSC6 and GPSC9. GPSC94, GPSC6 and GPSC9 predominantly contained VT serotypes, whereas GPSC5 had a lower proportion of vaccine types. Interestingly, not all globally dominant pneumococcal lineages were detected; only three lineages – GPSC1, GPSC6 and GPSC23 – were identified, representing 2.3%, 4.0% and 2.9% of the isolates, respectively, contributing a combined 9.2% of the overall collection.

Several GPSCs demonstrated strong associations with specific CCs. GPSC94, GPSC360, GPSC411, GPSC442, GPSC779 and GPSC904;9 correlated with CC2174, CC5537, CC9738, CC19166, CC19174 and CC63, respectively. In addition, GPSCs such as GPSC904;9, GPSC360, GPSC45 and GPSC5 contained both novel and known STs (Table S1). These findings highlight substantial genetic diversity of pneumococcal lineages in this population, with a significant representation of novel STs and non-vaccine type clusters, as well as revealing the dynamic nature of pneumococcal populations within global sequence clusters.

### Antibiotic resistance and determinant genes

All the study isolates were uniformly (100%) susceptible to LEV, LZD and VA. However, varying levels of non-susceptibility were observed for other antibiotics, including CHL (11%), COT (86.2%), ERY (8%), TET (59.7%), DA (5.2%) and PEN (34.5%). A strong correlation between the phenotypic and *in silico* resistance predictions was found for COT (93.5%), TET (89.7%), CHL (94.7%) and DA (77.8%). Among the isolates, 94 had all the PBP allele combinations identified and were predicted to be PEN susceptible. Most phenotypically non-susceptible pneumococci, however, had novel PBP allelic signatures.

**Fig. 3.**
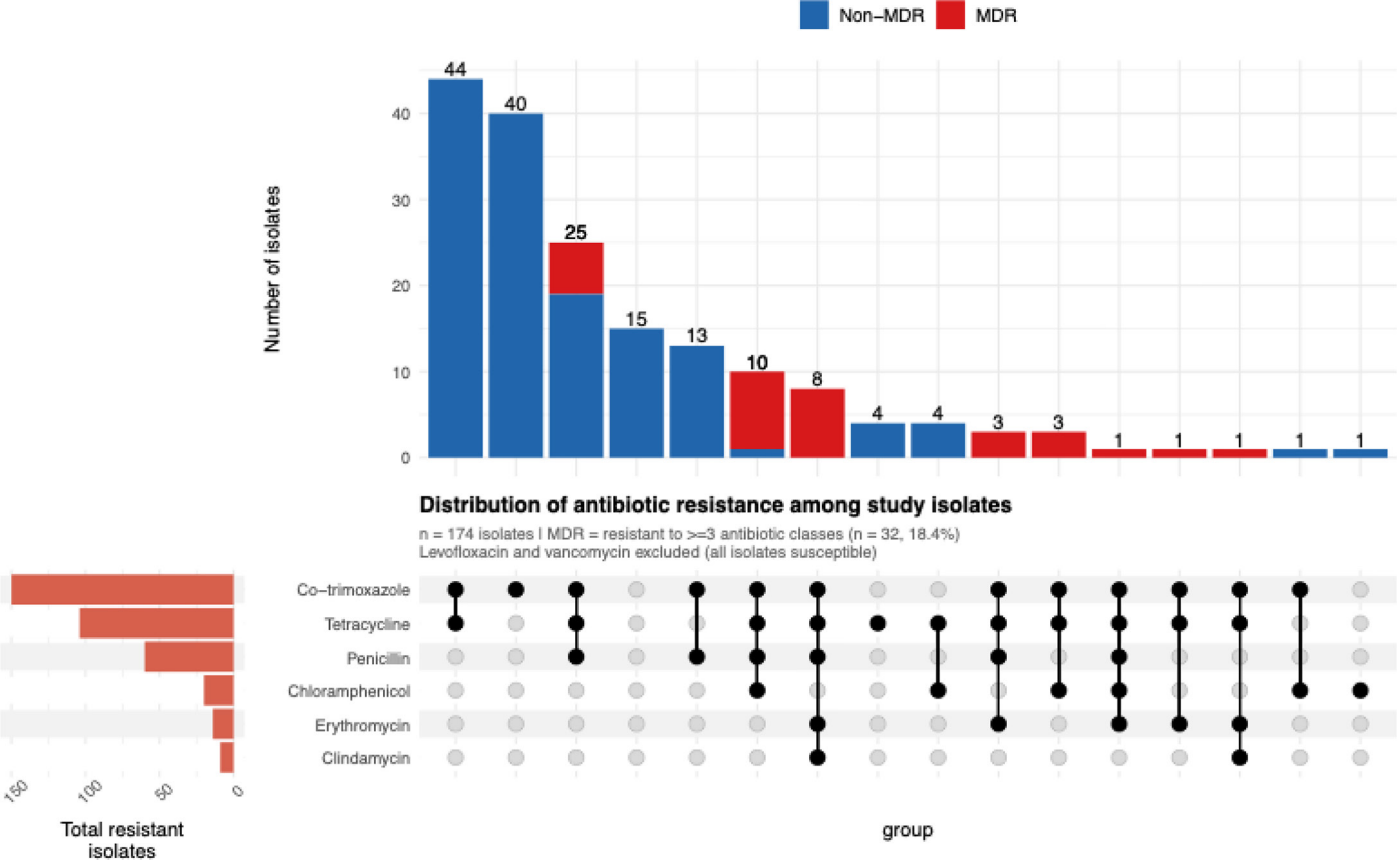
Distribution of antibiotic resistance among pneumococcal isolates (*n*=174).

Multidrug-resistant isolates accounted for 18.4% of the total isolates. Among these, VT isolates were significantly associated with MDR, comprising 34.3% of the MDR isolates (*P*=0.001; Table 2). Phenotypic resistance to CHL (17.9%), COT (86.2%), TET (73.1%) and PEN (53.7%) was significantly higher among the VT isolates (*P*=0.036; *P*=0.001; *P*=0.004; *P*=0.001, respectively, Table 2). Notably, all MDR isolates were also resistant to COT and TET (Fig. 3). Most (53%) of the MDR isolates exhibited non-susceptibility to PEN, COT and TET, with 23 isolates resistant to more than 3 antibiotics. The distribution and intersections of resistance patterns across antibiotic classes, stratified by MDR status, are shown in Fig. 3.

**Table 2.** Antibiotic susceptibility patterns of pneumococcal isolates (*N*=174) and distribution of resistance among vaccine types

	Resistance patterns			Vaccine types		
Antibiotics	Susceptible N (%)	Intermediate N (%)	Resistance N (%)	NVT N (%)	VT N (%)	*P*-value
CHL	154 (88.5)	0 (0)	20 (11.5)	8 (7.5)	12 (17.9)	0.036
COT	24 (13.8)	10 (5.7)	140 (80.5)	83 (77.6)	67 (100.0)	0.001
ERY	160 (92.0)	0 (0.0)	14 (8.0)	6 (5.6)	8 (11.9)	0.135
LEV	174 (100.0)	0 (0.0)	0 (0.0)	na	na	na
TET	70 (40.3)	7 (4.0)	97 (55.7)	55 (51.4)	49 (73.1)	0.004
VA	174 (100.0)	0 (0.0)	0 (0.0)	na	na	na
DA	165 (94.8)	0 (0.0)	9 (5.2)	4 (3.7)	5 (7.5)	0.28
LZD	174 (100.0)	0 (0.0)	0 (0.0)	na	na	na
PEN	114 (65.5)	58 (33.4)	2 (1.1)	24 (22.5)	36 (53.7)	0.001
MDR	142 (81.6)		32 (18.4)	9 (8.4)	23 (34.3)	0.001

MDR, multidrug resistance; NVT, non-vaccine types; VT, vaccine types (PCV13 serotypes).

**Fig. 4.**
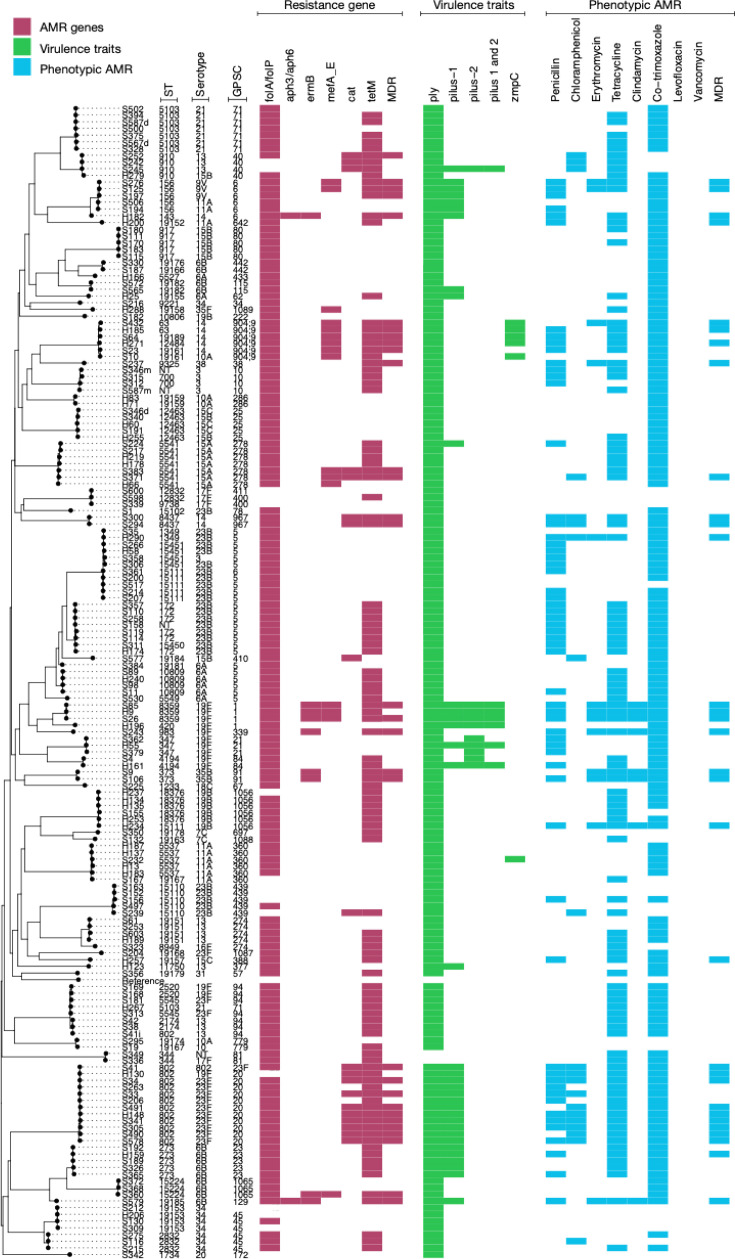
Maximum-likelihood tree constructed with all pneumococcal genomes in this study (*n*=174).

Genotypic resistance analysis revealed the presence of key resistance genes: *cat*, *tetM* and *ermB*, which confer resistance to CHL, TET and DA, respectively. These were detected in 19 (11%), 104 (60%) and 9 (5%) isolates, respectively. Phenotypic CHL resistance (17.9%) exceeded the rate of cat gene detection (11%), indicating incomplete concordance between phenotypic and genotypic methods. This discordance suggests that additional resistance mechanisms not captured by the gene panel – such as alternative *cat* variants or efflux-mediated resistance – may be contributing to phenotypic CHL resistance in a subset of isolates. Additional resistance genes, including *mefE*, *mefA*, *folA* and *folP*, were also identified. The genotypic resistance profiles of the isolates are detailed in Fig. 4.

MDR was strongly associated with specific STs, as shown in Fig. S2. All isolates belonging to ST63 (*n*=2), ST12484 (*n*=1), ST8437 (*n*=2), ST373 (*n*=2), ST9325 (*n*=1), ST983 (*n*=1), ST8359 (*n*=3), ST19152 (*n*=1), ST143 (*n*=1), 19185 (*n*=1) and ST19157 (*n*=1) were MDR. Additionally, multi-drug resistance was observed in isolates from ST1349 (*n*=1, 50%), ST273 (*n*=1, 20%), ST156 (*n*=2, 40%), ST5541 (*n*=1, 14.3%), ST802 (*n*=9, 69.2%) and ST15111 (*n*=2, 28.6%). Fifteen GPSCs were associated with MDR, with the most prevalent being GPSC1 (*n*=3, 75%), GPSC20 (*n*=9, 75%), GPSC6 (*n*=3, 42.9%) and GPSC9 (*n*=3, 50%) (Fig. 4).

Four PMEN (Pneumococcal Molecular Epidemiology Network) clones were identified: ST156 (Spain9V-3), ST273 (Greece6B-22), ST344 (NorwayNT-42) and ST63 (Sweden15A-25). Other closely related STs (locus variants) were also observed (Table S1). These findings highlight the significant diversity and widespread presence of MDR among the isolates.

### Virulence factors, phylogenetic and transmission analyses

Ply was detected in 173 of 174 isolates (99.4%), consistent with its near-universal distribution across *S. pneumoniae*. Given its high conservation and its role as a candidate antigen for next-generation protein-based vaccines, the broad prevalence of Ply across all GPSCs and serotypes in this collection supports its potential as a universal vaccine target. The presence of pilus islets was assessed based on specific gene markers. Pilus islet 1 (P1-1), identified through the presence of *rrgA/rrgB/rrgC*, was detected in 35 isolates (20%). Pilus islet 2 (PI-2), identified through the detection of *pitA* and *pitB*, was present in 10 isolates (6%). PI-1 was predominantly associated with GPSC1 (ST420/8359, serotype 19F), GPSC6 (ST156/143/15111, serotypes 14/11A/9V/23B), GPSC20 (ST802, serotypes 19F/23F) and GPSC23 (ST273, serotype 6B), though it was occasionally observed in other GPSCs. PI-2 was primarily linked to GPSC1 (ST420/8359, serotype 19F) and GPSC21 (ST347, serotype 19F).

The combination of PI-1 and PI-2 was detected in four GPSCs: GPSC1 (100%, *n*=4), GPSC21 (33%, *n*=1), GPSC40 (25%, *n*=1) and GPSC84 (50%, *n*=1) (Fig. 4 and Table S1). These findings suggest that the pilus islets are variably distributed across the isolates, with PI-1 being more common than PI-2 or their combination. The *zmpC* gene, a virulence factor associated with zinc metalloproteinase, was identified in six isolates (3.4%). Among these, one isolate belonged to GPSC360 (*n*=1, 17%), while the remaining five were associated with GPSC9 (*n*=5, 83%). This may indicate that *zmpC* is relatively rare but shows clustering within specific GPSCs.

**Fig. 5.**
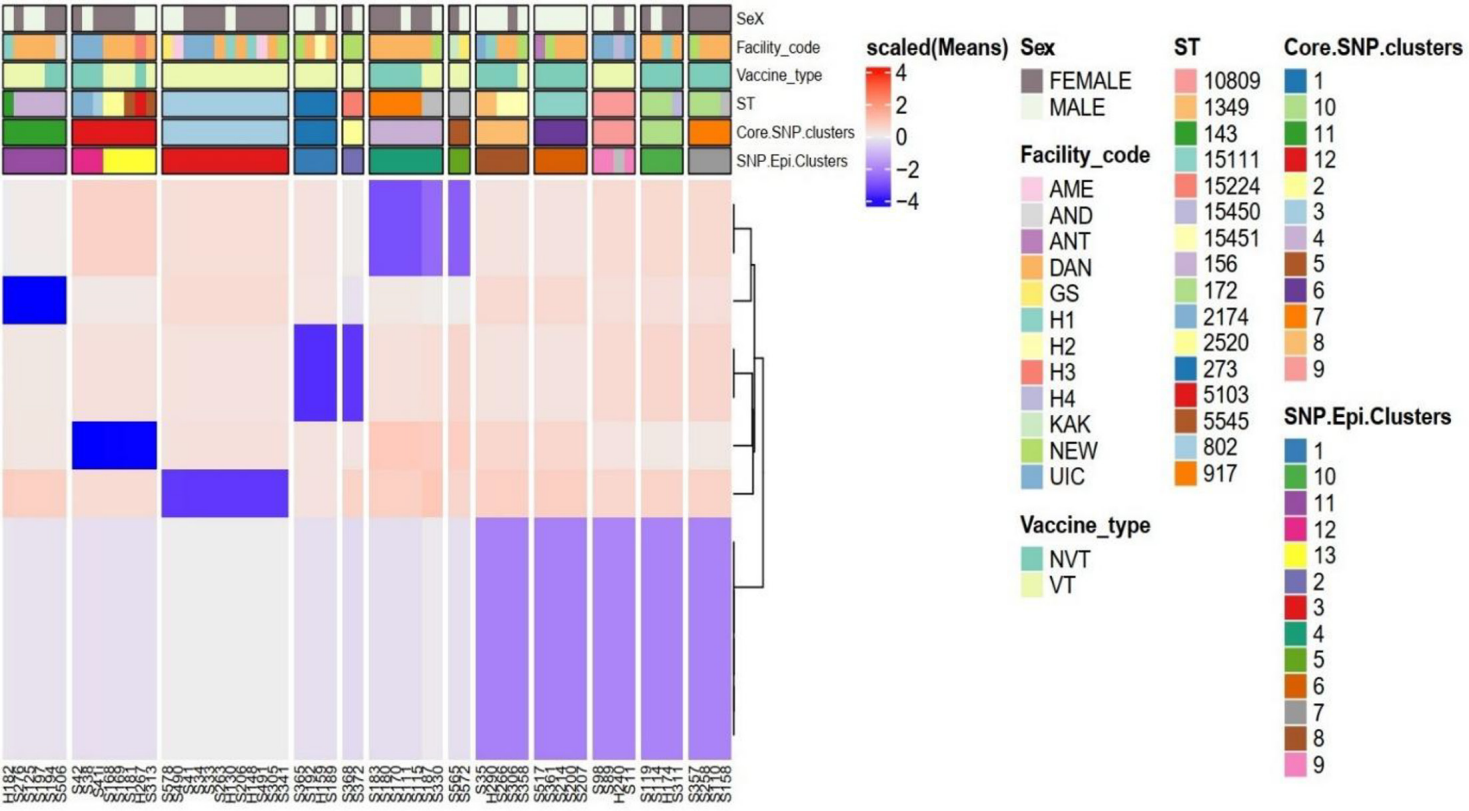
Summary of the transmission network and cluster analysis based on K-means.

Phylogenetic analysis incorporating core-SNP clustering and SNP-Epi clustering identified 12 distinct strain-sharing clusters comprising 63 pneumococcal isolates. Isolates within each cluster were closely genetically related (≤12–13 core-genome SNPs), even though they were not limited to specific facilities (Fig. 5). Genetically related strains were detected across both pre-school settings (DAN and UIC) and child welfare clinics, despite their geographic separation within Cape Coast (Fig. S3). Details of the serotypes, GPSCs and MDR status represented within each cluster are provided in Table S1. We note that genetic relatedness and strain sharing do not constitute direct evidence of transmission; our cross-sectional study design does not allow formal attribution of shared lineages to specific transmission events or routes.

## Discussion

This study investigates the genomic characteristics of the *S. pneumoniae* population isolated from healthy children under five in Ghana. While previous studies have primarily focused on phenotypic analyses, this genomic investigation expands the understanding of STs, GPSCs and antimicrobial resistance determinants. These insights provide a more comprehensive framework for monitoring vaccine impact and understanding the dynamics within the pneumococcal population. Carriage studies are particularly valuable in populations where *S. pneumoniae* prevalence is high, as they offer an accurate representation of circulating pneumococcal serotypes [34]. The nasopharynx supports the co-colonization of multiple bacterial species and pneumococcal strains. Following PCV13 introduction, the removal of dominant vaccine-type lineages through immune pressure may expand the available colonization niche for non-vaccine type (NVT) strains – a phenomenon of competitive release – facilitating the expansion of pneumococcal clones that are both competent for colonization and capable of causing infections. This underscores the importance of genomic-level surveillance in informing public health interventions.

The near-perfect concordance between phenotypic serotyping and genotypic serotyping observed in this study underscores the utility of WGS as a reliable tool for pneumococcal serotyping. While the serotypes covered by PCV-13 persisted at 38.5%, their prevalence was markedly lower compared to the pre-vaccination era, as reported in previous studies [22,23]. This decline suggests a vaccine escape mechanism exhibited by certain serotypes, such as serotype 3, which remain detectable despite vaccination efforts. The persistence of serotype 3 may be attributed to the low efficacy of the antibody-mediated immune response, characterized by reduced levels of anti-serotype 3 capsular polysaccharide antibodies, leading to impaired opsonophagocytosis in susceptible individuals [35]. For other persistent VT serotypes observed in this study – including 6B, 19F, 6A and 23F – the picture is likely multifactorial. Incomplete vaccine coverage is a plausible contributor: although national PCV13 coverage in Ghana has been estimated at ~97%, sub-national variation and the absence of a booster dose in the primary schedule (6–10–14 weeks) may leave pockets of susceptibility in some communities [14]. Additional factors include natural waning of vaccine-induced antibody titres, which may be particularly relevant for serotypes where responses decline more rapidly post-immunization; the relatively recent timeline of PCV13 introduction in Ghana (2012), which may not yet have generated maximal herd immunity across all age groups; and the fact that PCV13 reduces but does not eliminate nasopharyngeal carriage in immunized children, meaning vaccinated individuals may still harbour and transmit VT strains [36,37]. Older unvaccinated children and adults may further sustain VT circulation as community reservoirs [38].

In contrast, NVTs were highly prevalent, accounting for 61.5% of isolates. This finding aligns with reports of a gradual shift in pneumococcal dissemination patterns and serotype replacement [39]. NVTs such as 23B and 15B, which were highly represented in this study, have also been implicated in invasive pneumococcal diseases in Ghana and Germany [40].

To address the emergence and expansion of NVTs, newer vaccines such as PCV-15 and PCV-20 have been introduced for immunization programmes. PCV-20, which includes PCV-13 serotypes along with 10A, 11A and 15B, covered 52.3% of the serotypes detected in this study. In contrast, PCV-15, which includes PCV-13 plus 22F, 33, had the same coverage as PCV-13, as neither 22F nor 33F was detected in this study.

This study reveals a locally diverse population of *S. pneumoniae*, characterized by a high number of well-documented and newly emerged STs. In agreement with a recent report by Mills *et al*. [41], which identified five newly emerged STs geographically limited to Ghana (ST15489, ST15459, ST15461, ST15448 and ST1511), we observed significant diversity, including ST802 which intriguingly expressed multiple serotypes (19F, 23F and 13). Similar patterns of capsular switching were observed in other STs, such as ST156 (9V/11A), ST15451 (23B/3), ST19161 (10A/14), ST910 (15B/13), ST15111 (15B/19B) and ST344 (NT/17F). Capsular switching, a mechanism of immune evasion that enables *S. pneumoniae* to escape host antibodies by exchanging the *cps* locus, is driven by evolutionarily conserved genetic recombination processes and is further influenced by vaccine- and antibiotic-induced selective pressures [42].

This study identified several PMEN clones, which are of public health concern due to their global spread, disease-causing potential, vaccine escape mechanisms and association with antibiotic resistance. The PMEN clones identified included ST156 (PMEN 3), associated with PEN resistance, and ST344 (PMEN42). Additionally, ST8359 and ST983, triple-locus variants of Taiwan19F-14 (PMEN14), were found to exhibit MDR, including high resistance to ERY, CHL and TET. Novel STs such as ST19157, ST19185 (a double-locus variant of Maryland6B-17) and ST19152 were also associated with MDR. While there was strong concordance between MLST and GPSCs, some STs – such as ST8359, ST1349, ST15111, ST1540, ST15451, ST9221, ST15102, ST802, ST2520, ST1734, ST9738 and ST15110 – did not align with their expected GPSCs according to the GPSC-ST lookup table (accessed 1 October 2024).

Whole genome clustering (as defined in the **Methods**), which accounts for genetic variation across the entire genome [43], revealed a greater representation of non-vaccine type (NVT) lineages compared to vaccine type (VT) lineages, with only four GPSCs expressing both serotype groups. This shift towards NVT lineages may be attributed to the expansion of NVT serotypes into niches vacated by VT lineages, as well as capsular switching [17,19]. Notably, GPSC5 was the most prevalent lineage, associated with serotypes 3, 6A and 23B. This lineage has been reported globally to exhibit diverse serotypes, including serotype 35B/D in South Africa [44,45], 19A in Argentina [46], 23A in Canada [47] and 23B in Australia [48]. However, in this study, GPSC5 expressed serotype 3, contrasting with findings from other regions where serotype 3 is typically associated with GPSC12 [19,48].

GPSC21, a VT lineage detected in East Africa (Malawi and Mozambique) [49], was also reported in this study and was found to be associated with both P1-1 and P1-2 pilus islets. GPSC6, a globally recognized lineage known to adapt under vaccine pressure, expressed multiple serotypes (14/11A/9V/23B) from STs such as ST156, ST143 and ST15111 and exhibited non-susceptibility to PEN, TET and COT. The presence of PI-1 in GPSC6 likely contributes to its propensity to cause infections [50].

The study further confirmed that vaccine serotypes are linked to MDR, with GPSC1, GPSC9 and GPSC10 contributing to antibiotic resistance (Table 2). However, in this study, only GPSC9 was exhibited, in contrast to previous reports of GPSC1 and GPSC10 being associated with MDR [51]. The pilus genes associated with GPSC9 and GPSC10 may contribute to resistance [25], a phenomenon also observed in GPSC10 in Sweden [51]. GPSC9 was linked to the *zmpC* gene, which encodes zinc metalloproteinase C and was carried by serotype 14 in this study. This contrasts with findings in Italy, where *zmpC* is carried by serotypes 8/11A [52], and in the Netherlands, where it is associated with serotypes 8/4/33 A/F/11A/D [53]. Finally, all isolates belonging to GPSC20 exhibited resistance to CHL, further emphasizing the diverse resistance patterns within pneumococcal populations.

Ply, pili and zmpC are highly relevant, conserved antigens being investigated for the development of next-generation, protein-based vaccines against *S. pneumoniae*. While current vaccines target the polysaccharide capsule, these protein antigens offer the potential for broader, serotype/GPSC-independent and, in some cases, mucosal protection [7]. Ply is present in virtually all pneumococcal strains, making it a target for a universal vaccine, whereas pili (particularly PI-1) and ZmpC are found among a subset of strains, with PI-1 pili predominantly associated with vaccine serotypes in the pre-PCV era [54]. Of these candidates, only a trivalent formulation combining detoxified Ply (PlyD1), PhtD and PcpA has reached clinical evaluation, demonstrating safety and immunogenicity in a phase I study [55].

*S. pneumoniae* is spread primarily through direct contact with respiratory droplets from infected persons or asymptomatic carriers. Factors such as overcrowding and young age influence pneumococcal transmission. In Cape Coast, the majority of preschool children are transported in school buses from one location to another. These buses sometimes transport children between different locations and schools (Fig. S3). These shared travel routes represent plausible opportunities for exposure to common pneumococcal lineages, and this may explain the detection of genetically related strains across different facilities in our study (Fig. 5). Isolates within the identified strain-sharing clusters share at most 13 core-genome SNPs, consistent with recent common ancestry; however, genetic relatedness alone does not constitute direct evidence of transmission, and our cross-sectional design precludes the formal identification of transmission chains. Notably, one cluster of four strains (H159, S365, S189 and S192) shares lineages at only one SNP despite originating from different facilities within the same geographic location, suggesting a high degree of recent genetic relatedness that warrants further longitudinal investigation.

## Conclusion

This study provides a comprehensive characterization of the *S. pneumoniae* population in healthy children under five in Ghana using WGS. The persistence of vaccine-type serotypes was observed 6 years after the introduction of PCV-13, highlighting ongoing selective pressures. In addition, the identification of novel STs and GPSC lineages unique to Ghana underscores the high genetic diversity within the pneumococcal population.

Although not all globally disseminated GPSCs were detected, GPSC5 emerged as a predominant lineage driving multiple serotypes, including serotypes 3, 6A and 23B. The study also identified GPSCs of public health concern, emphasizing the importance of continuous surveillance to track their evolution and impact. They also provide crucial insight to guide future vaccination strategies to control pneumococcal disease. Furthermore, AMR databases should be updated to account for potentially resistant isolates harbouring novel resistance alleles, ensuring robust mechanisms to track and combat AMR in pneumococcal populations.

## Supplementary material

10.1099/mgen.0.001734 Supplementary Material 1.

## Abbreviations

WGS: whole-genome sequencing
GPSCs: Global Pneumococcal Sequence Clusters
CCs: clonal complexes
STs: sequence types
mPCR: multiplex PCR
CHL: chloramphenicol
COT: cotrimoxazole
DA: clindamycin
ERY: erythromycin
LEV: levofloxacin
LZD: linezolid
OX: oxacillin
PEN: penicillin
TET: tetracycline
VA: vancomycin
MDR: multidrug resistance
PCVs: pneumococcal conjugate vaccines
PCV7: 7-valent PCV
PCV-10: 10-valent PCV
PCV-13: 13-valent PCV
P1-1: pilus islet 1
PI-2: pilus islet 2
Ply: pneumolysin
zmpC: zinc metalloproteinase C
RCH: reproductive and child health

## References

[1] Centers for Disease Control and Prevention (2021). Current epidemiology of pneumococcal disease and pneumococcal vaccine coverage among adults, United States. https://stacks.cdc.gov/view/cdc/107067.

[2] Wang G-M, Tao W, Pang X-Y, Xin Y, Gou Z-H (2025). Global, regional, and national burden of pneumococcal disease among children and adolescents aged <20 years from 1990 to 2021: a predictive analysis. Front Public Health.

[3] Wahl B, O’Brien KL, Greenbaum A, Majumder A, Liu L (2018). Burden of *Streptococcus pneumoniae* and *Haemophilus influenzae* type b disease in children in the era of conjugate vaccines: global, regional, and national estimates for 2000-15. Lancet Glob Health.

[4] Jiang SM, Wang L, Reeves PR (2001). Molecular characterization of *Streptococcus pneumoniae* type 4, 6B, 8, and 18C capsular polysaccharide gene clusters. Infect Immun.

[5] Yu J, Ravenscroft N, Davey P, Liyanage R, Lorenz O (2025). New pneumococcal serotype 20C is a WciG O-acetyltransferase deficient variant of canonical serotype 20B. *Microbiol Spectr*.

[6] Narciso AR, Dookie R, Nannapaneni P, Normark S, Henriques-Normark B (2025). *Streptococcus pneumoniae* epidemiology, pathogenesis and control. *Nat Rev Microbiol*.

[7] Kadioglu A, Weiser JN, Paton JC, Andrew PW (2008). The role of *Streptococcus pneumoniae* virulence factors in host respiratory colonization and disease. *Nat Rev Microbiol*.

[8] Geno KA, Gilbert GL, Song JY, Skovsted IC, Klugman KP (2015). Pneumococcal capsules and their types: past, present, and future. *Clin Microbiol Rev*.

[9] Abramson JS, Baker CJ, Fisher MC, Gerber MA, Meissner HC (2000). Policy statement: recommendations for the prevention of pneumococcal infections, including the use of pneumococcal conjugate vaccine (Prevnar), pneumococcal polysaccharide vaccine, and antibiotic prophylaxis. Pediatrics.

[10] O’Brien KL, Wolfson LJ, Watt JP, Henkle E, Deloria-Knoll M (2009). Burden of disease caused by *Streptococcus pneumoniae* in children younger than 5 years: global estimates. *Lancet*.

[11] van Hoek AJ, Andrews N, Waight PA, Stowe J, Gates P (2012). The effect of underlying clinical conditions on the risk of developing invasive pneumococcal disease in England. *J Infect*.

[12] Whitney CG, Goldblatt D, O’Brien KL (2014). Dosing schedules for pneumococcal conjugate vaccine. Pediatr Infect Dis J.

[13] Madhi SA, Nunes MC (2016). The potential impact of pneumococcal conjugate vaccine in Africa: considerations and early lessons learned from the South African experience. Hum Vaccin Immunother.

[14] Ibrahim AM, Owusu R, Nonvignon J (2024). Sustainability of pneumococcal conjugate vaccination in Ghana: a cost-effectiveness analysis in the context of donor transition. Front Public Health.

[15] Brandileone M-CC, Almeida SCG, Bokermann S, Minamisava R, Berezin EN (2021). Dynamics of antimicrobial resistance of Streptococcus pneumoniae following PCV10 introduction in Brazil: Nationwide surveillance from 2007 to 2019. *Vaccine*.

[16] Andam CP, Hanage WP (2015). Mechanisms of genome evolution of *Streptococcus*. Infect Genet Evol.

[17] Weinberger DM, Malley R, Lipsitch M (2011). Serotype replacement in disease after pneumococcal vaccination. *Lancet*.

[18] Lewnard JA, Hanage WP (2019). Making sense of differences in pneumococcal serotype replacement. Lancet Infect Dis.

[19] Almeida SCG, Lo SW, Hawkins PA, Gladstone RA, Cassiolato AP (2021). Genomic surveillance of invasive *Streptococcus pneumoniae* isolates in the period pre-PCV10 and post-PCV10 introduction in Brazil. *Microb Genom*.

[20] Denno DM, Frimpong E, Gregory M, Steele RW (2002). Nasopharyngeal carriage and susceptibility patterns of *Streptococcus pneumoniae* in Kumasi, Ghana. West Afr J Med.

[21] Mills RO, Twum-Danso K, Owusu-Agyei S, Donkor ES (2015). Epidemiology of pneumococcal carriage in children under five years of age in Accra, Ghana. Infect Dis.

[22] Dayie NTKD, Arhin RE, Newman MJ, Dalsgaard A, Bisgaard M (2013). Penicillin resistance and serotype distribution of *Streptococcus pneumoniae* in Ghanaian children less than six years of age. BMC Infect Dis.

[23] Dayie NTKD, Tettey EY, Newman MJ, Bannerman E, Donkor ES (2019). Pneumococcal carriage among children under five in Accra, Ghana, five years after the introduction of pneumococcal conjugate vaccine. BMC Pediatr.

[24] Narwortey DK, Owusu-Ofori A, Slotved H-C, Donkor ES, Ansah PO (2021). Nasopharyngeal carriage of *Streptococcus pneumoniae* among healthy children in Kassena-Nankana districts of Northern Ghana. BMC Infect Dis.

[25] Mills RO, Abdullah MR, Akwetey SA, Sappor DC, Cole I (2020). Post-vaccination *Streptococcus pneumoniae* carriage and virulence gene distribution among children less than five years of age, Cape Coast, Ghana. Microorganisms.

[26] Satzke C, Turner P, Virolainen-Julkunen A, Adrian PV, Antonio M (2013). Standard method for detecting upper respiratory carriage of *Streptococcus pneumoniae*: updated recommendations from the World Health Organization Pneumococcal Carriage Working Group. *Vaccine*.

[27] Magiorakos A-P, Srinivasan A, Carey RB, Carmeli Y, Falagas ME (2012). Multidrug-resistant, extensively drug-resistant and pandrug-resistant bacteria: an international expert proposal for interim standard definitions for acquired resistance. *Clin Microbiol Infect*.

[28] Lees JA, Harris SR, Tonkin-Hill G, Gladstone RA, Lo SW (2019). Fast and flexible bacterial genomic epidemiology with PopPUNK. Genome Res.

[29] Epping L, van Tonder AJ, Gladstone RA, Bentley SD, Page AJ (2018). SeroBA: rapid high-throughput serotyping of *Streptococcus pneumoniae* from whole genome sequence data. Microb Genom.

[30] Sherry NL, Horan KA, Ballard SA, Gonҫalves da Silva A, Gorrie CL (2023). An ISO-certified genomics workflow for identification and surveillance of antimicrobial resistance. *Nat Commun*.

[31] Croucher NJ, Page AJ, Connor TR, Delaney AJ, Keane JA (2015). Rapid phylogenetic analysis of large samples of recombinant bacterial whole genome sequences using Gubbins. *Nucleic Acids Res*.

[32] Kwenda S, Shuping L, Mashau R, Ismail H, Govender NP (2025). SNP2Cluster: a core SNP and k-means clustering-based tool for enhanced transmission cluster detection in outbreak scenarios. Zenedo.

[33] Nascimento M, Sousa A, Ramirez M, Francisco AP, Carriço JA (2017). PHYLOViZ 2.0: providing scalable data integration and visualization for multiple phylogenetic inference methods. *Bioinformatics*.

[34] Donkor ES (2013). Molecular typing of the pneumococcus and its application in epidemiology in sub-Saharan Africa. Front Cell Infect Microbiol.

[35] Oliveira GS, Rivera J, Rodrigues TC, Carneiro GB, Ribeiro OG (2024). Serotype 3 *Streptococcus pneumoniae* escapes the immune responses induced by PCV13 in mice with high susceptibility to infection. Immun Inflamm Dis.

[36] Janapatla RP, Hsu M-H, Chen C-L, Wei S-H, Yu M-J (2020). Persistence of immunity in children immunised with 13-valent pneumococcal conjugate vaccine and impact on nasopharyngeal carriage: a cross-sectional study. Thorax.

[37] Dagan R, Juergens C, Trammel J, Patterson S, Greenberg D (2015). Efficacy of 13-valent pneumococcal conjugate vaccine (PCV13) versus that of 7-valent PCV (PCV7) against nasopharyngeal colonization of antibiotic-nonsusceptible *Streptococcus pneumoniae*. *J Infect Dis*.

[38] Flasche S, Lipsitch M, Ojal J, Pinsent A (2020). Estimating the contribution of different age strata to vaccine serotype pneumococcal transmission in the pre vaccine era: a modelling study. BMC Med.

[39] Obolski U, Lourenço J, Thompson C, Thompson R, Gori A (2018). Vaccination can drive an increase in frequencies of antibiotic resistance among nonvaccine serotypes of *Streptococcus pneumoniae*. *Proc Natl Acad Sci USA*.

[40] van der Linden M, Perniciaro S, Imöhl M (2015). Increase of serotypes 15A and 23B in IPD in Germany in the PCV13 vaccination era. BMC Infect Dis.

[41] Mills RO, Abdullah MR, Akwetey SA, Sappor DC, Gámez G (2022). Molecular epidemiology of multidrug-resistant pneumococci among Ghanaian children under five years post PCV13 using MLST. Microorganisms.

[42] Lo SW, Gladstone RA, van Tonder AJ, Lees JA, du Plessis M (2019). Pneumococcal lineages associated with serotype replacement and antibiotic resistance in childhood invasive pneumococcal disease in the post-PCV13 era: an international whole-genome sequencing study. Lancet Infect Dis.

[43] Gladstone RA, Lo SW, Lees JA, Croucher NJ, van Tonder AJ (2019). International genomic definition of pneumococcal lineages, to contextualise disease, antibiotic resistance and vaccine impact. *EBioMedicine*.

[44] Lo SW, Hawkins PA, Jibir B, Hassan-Hanga F, Gambo M (2023). Molecular characterization of *Streptococcus pneumoniae* causing disease among children in Nigeria during the introduction of PCV10 (GSK). Microb Genom.

[45] Ndlangisa KM, du Plessis M, Lo S, de Gouveia L, Chaguza C (2022). A *Streptococcus pneumoniae* lineage usually associated with pneumococcal conjugate vaccine (PCV) serotypes is the most common cause of serotype 35B invasive disease in South Africa, following routine use of PCV. Microb Genom.

[46] Gagetti P, Lo SW, Hawkins PA, Gladstone RA, Regueira M (2021). Population genetic structure, serotype distribution and antibiotic resistance of *Streptococcus pneumoniae* causing invasive disease in children in Argentina. Microb Genom.

[47] Golden AR, Adam HJ, Baxter M, Martin I, Demczuk W (2021). Whole genome characterization of *Streptococcus pneumoniae* from respiratory and blood cultures collected from Canadian hospitals before and after PCV-13 implementation in Canada: focus on serotypes 22F and 33F from CANWARD 2007-2018. *Vaccine*.

[48] Higgs C, Kumar LS, Stevens K, Strachan J, Sherry NL (2023). Population structure, serotype distribution and antibiotic resistance of *Streptococcus pneumoniae* causing invasive disease in Victoria, Australia. Microb Genom.

[49] Javaid N, Olwagen C, Nzenze S, Hawkins P, Gladstone R (2022). Population genomics of pneumococcal carriage in South Africa following the introduction of the 13-valent pneumococcal conjugate vaccine (PCV13) immunization. Microb Genom.

[50] Egorova E, Kumar N, Gladstone RA, Urban Y, Voropaeva E (2022). Key features of pneumococcal isolates recovered in Central and Northwestern Russia in 2011-2018 determined through whole-genome sequencing. Microb Genom.

[51] Yamba Yamba L, Uddén F, Fuursted K, Ahl J, Slotved H-C (2022). Extensive/multidrug-resistant pneumococci detected in clinical respiratory tract samples in southern Sweden are closely related to international multidrug-resistant lineages. Front Cell Infect Microbiol.

[52] Camilli R, Daprai L, Cavrini F, Lombardo D, D’Ambrosio F (2013). Pneumococcal carriage in young children one year after introduction of the 13-valent conjugate vaccine in Italy. *PLoS One*.

[53] Cremers AJH, Kokmeijer I, Groh L, de Jonge MI, Ferwerda G (2014). The role of ZmpC in the clinical manifestation of invasive pneumococcal disease. *Int J Med Microbiol*.

[54] Barocchi MA, Ries J, Zogaj X, Hemsley C, Albiger B (2006). A pneumococcal pilus influences virulence and host inflammatory responses. *Proc Natl Acad Sci USA*.

[55] Brooks WA, Chang LJ, Sheng X, Hopfer R, Bruyn G (2015). Safety and immunogenicity of a trivalent recombinant PcpA, PhtD, and PlyD1 pneumococcal protein vaccine in adults, toddlers, and infants: a phase I randomized controlled study. *Vaccine*.

