# Supplementary material for: Genomic surveillance of post-immunization pneumococcal carriage in Ghana
Source: Microb Genom. 2026 Jun 17;12(6):001734. doi: 10.1099/mgen.0.001734 (PMC13274843; doi:10.1099/mgen.0.001734)
Supplement: Supplementary Material 1. [file mgen-12-01734-s001.pdf]

Table S1. Relationship between GPSCs, Clonal Complex, Sequence Types and Serotypes

GPSC	Clonal complex	Sequence Type	PMEN clone	Serotype	Serotype group (VT/NVT)
1	Singleton	420		19F	VT
	Singleton	8359	TLV-Taiwan19F-14		
5	172	172	DLV-S.Africa19A-7<br>TLV-Hungary19A-6	3	VT
		15450			
	Singleton	5549		6A	VT
	Singleton	10809			
	1349	15111			
		1349	DLV-Colombia23F-26	23B	NVT
		15451		23B, 3	NVT, VT
	Singleton	**19181**			
6	Singleton	143	DLV-Spain9V-3	14	VT
	Singleton	156	**Spain9V-3**	11A, 9V	NVT, VT
	Singleton	15111		23B	NVT
10	Singleton	700	DLV-Denmark14-32	3	VT
	Singleton	NA		3	VT
20	Singleton	802		23F, 19F	VT
21	Singleton	347		19F	VT
23	Singleton	273	**Greece6B-22**	6B	VT
25	Singleton	12463		15B, 15C	NVT
34	Singleton	9221		34	NVT
38	Singleton	9325		38	NVT
40	Singleton	910		15B, 13	NVT
45	Singleton	2825		34	NVT
	Singleton	**19153**			
57	Singleton	**19178**		31	
62	Singleton	**19155**		6A	VT
67	Singleton	1233		18C	VT

71	Singleton	5103		21	NVT
78	Singleton	15102		23B	NVT
80	Singleton	917		15B	NVT
81	Singleton	344	**NorwayNT-42**	NT, 17F	NVT
84	Singleton	4194		19F	VT
91	Singleton	373		35B	NVT
94	Singleton	802		13, 19F, 23F	NVT
	2174	2174		19F	VT
		2520			
		5545		23F	VT
115	Singleton	**19182**		6B	VT
129	Singleton	**19185**	DLV-Maryland6B-17	6B	VT
172	Singleton	1734		20	NVT
222	Singleton	10806		19B	NVT
274	Singleton	8949		16F	NVT
278	Singleton	5541		15A	NVT
286	Singleton	**19159**		10A	NVT
339	Singleton	983	TLV-Taiwan19F-14	19F	VT
360	5537	5537		11A	NVT
		**19164**			
377	Singleton	11750		13	NVT
388	Singleton	**19157**		15C	NVT
410	Singleton	**19184**		15B	NVT
411	9738	9738		17F	NVT
		12832			
433	Singleton	5527		6A	VT
439	Singleton	15110		23B	NVT
442	19166	**19166**		6B	VT
		**19176**			
642	Singleton	**19152**		11A	
779	19167	**19174**		6B	VT

		**19167**			
967	Singleton	8437		14	VT
904;9	63	63	**Sweden15A-25**	14	VT
		12484	SLV-Sweden15A-25		
		**19161**	SLV-Sweden15A-25	10A, 14	NVT
		**19189**	DLV- Sweden15A-25	14	VT
1088	Singleton	**19163**		7C	NVT
1056	Singleton	15111		19B, 23B	NVT
	Singleton	18376		19B	NVT
1065	Singleton	15224		6B	VT
1086	Singleton	NA			
1087	Singleton	**19168**		23F	VT
1089	Singleton	**19158**		35F	NVT

GPSCs: Global Pneumococcal Sequence Clusters; Sequence types in **bold** are novel sequence types in this study; NA: Sequence types not typed due to truncation; NVT: non-vaccine types (non-PCV13 serotypes); vaccine types (PCV13 serotypes). Clonal complexes are in red.

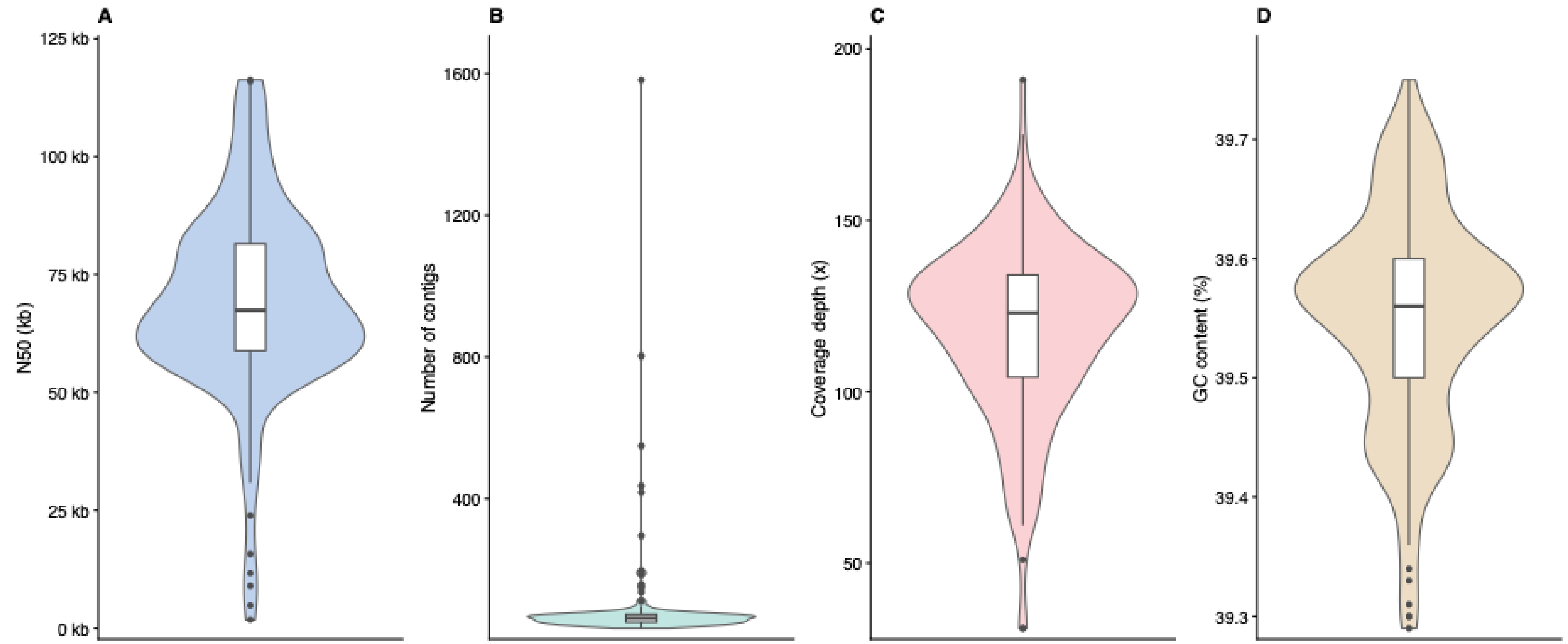

Figure S1. Assembly quality control metrics for the 174 *Streptococcus pneumoniae* genomes included in this study. For each metric, the violin plot shows the full distribution of values across all assemblies, and the embedded box plot shows the median (centre line), interquartile range (box), and 1.5× IQR (whiskers); individual points beyond the whiskers represent outliers. (**A**) N50 value (kb): the sequence length below which 50% of the total assembly length is contained, reflecting assembly contiguity. The y-axis starts at zero; all N50 values are positive. (**B**) Number of contigs per assembly, reflecting fragmentation. (**C**) Mean sequencing coverage depth (×). (**D**) GC content (%), reflecting nucleotide composition of the assembled genome.

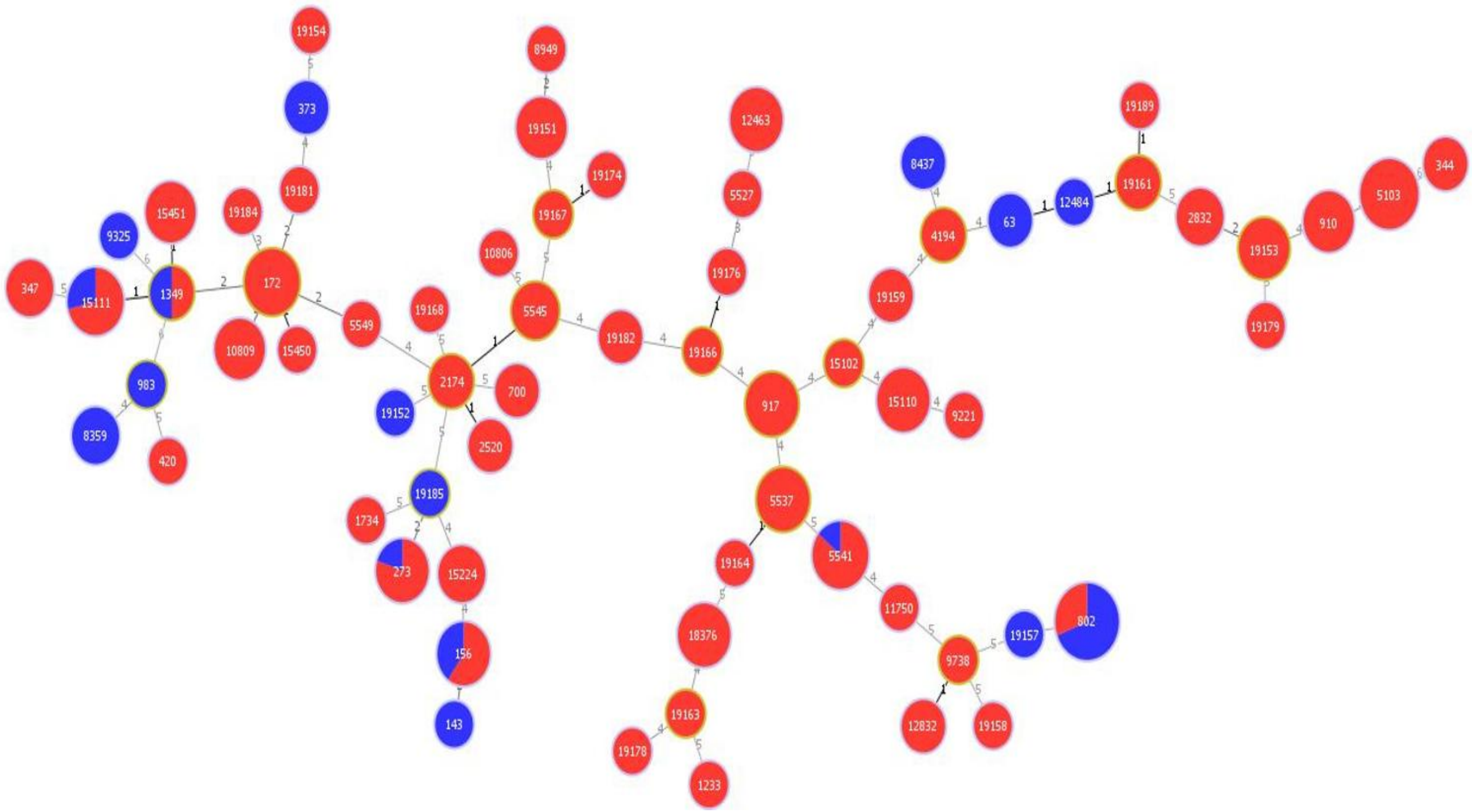

Figure S2. Genetic Relationships of Sequence Types (STs) of *S. pneumoniae* isolates in this study. Minimum spanning tree was constructed using Phyloviz software by the eBURST algorithm. The numbers represent the sequence types, and the size of the circles indicates the number of isolates belonging to a sequence type. The numbers on the lines represent the differences in loci between two STs. Multidrug resistance = blue and red = not multidrug resistance.

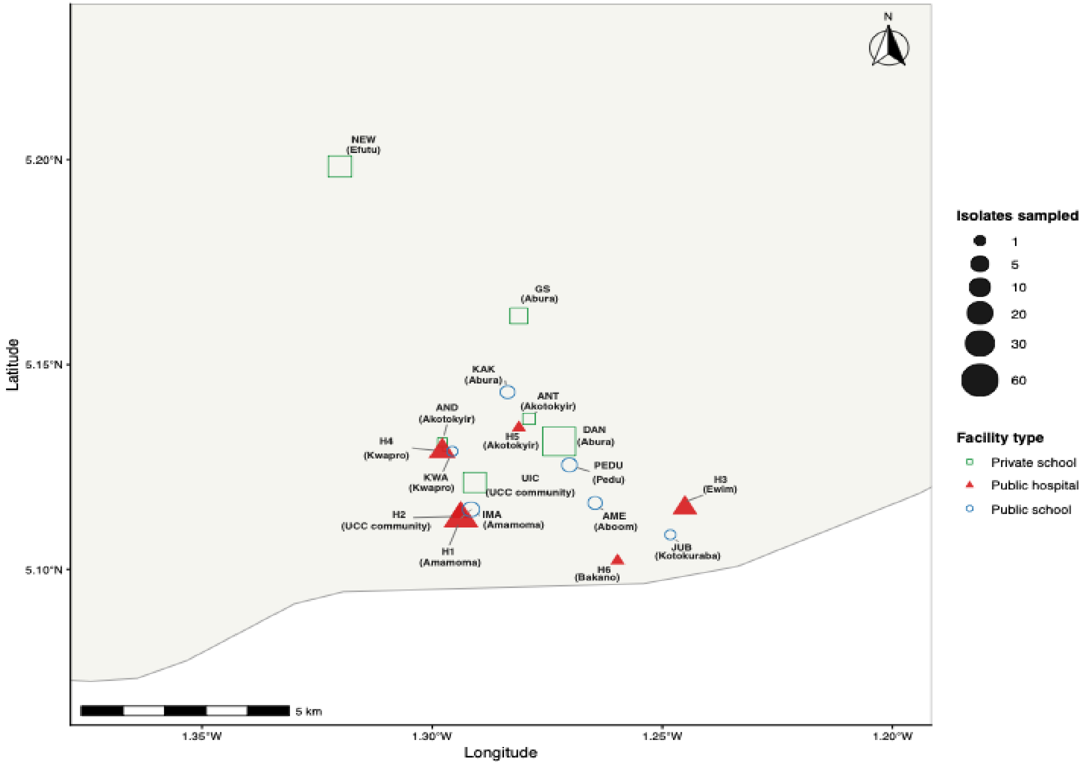

Figure S3. **Geographic distribution of pneumococcal carriage sampling sites in Cape Coast Metropolitan Area, Ghana.** Each symbol represents one study facility (n = 18 total: 6 public hospitals, 6 public schools, 6 private schools). Symbol shape and colour indicate facility type: red triangles, public hospitals; blue circles, public schools; green squares, private schools. Symbol size is proportional to the number of pneumococcal isolates collected at that facility. Facility codes are labelled with connecting lines where labels would otherwise overlap. Three pairs/groups of facilities share the same geographic location (IMA with H1; KWA and AND with H4; ANT with H5), reflecting schools situated on or immediately adjacent to hospital grounds; these co-located facilities are displayed with a small positional offset so that all symbols are individually visible, with dashed lines indicating their true shared geographic position. All 174 isolates included in the study were collected within a three-month window (January–March 2018).
